# The relationship between objective and subjective social isolation and well-being: a national cross-sectional survey in Wales

**DOI:** 10.3389/fpubh.2026.1918382

**Published:** 2026-10-13

**Authors:** Carys Dale, Sara Wood, Catherine A. Sharp, Karen Hughes

**Affiliations:** 1 Policy and International Health Directorate, World Health Organization Collaborating Centre on Investment for Health & Well-Being, Public Health Wales, Cardiff, United Kingdom; 2 School of Health Sciences, Bangor University, Wrexham, United Kingdom

**Keywords:** loneliness, population, social isolation, socio-demographics, well-being

## Abstract

**Introduction:**

Social isolation is increasingly recognised as a public health issue with wide ranging impacts on health and well-being. However, research examining links with health and well-being often focuses on subjective or objective forms of isolation without considering combined associations.

**Methods:**

Data were collected through a national public health panel survey in Wales, UK (*n* = 2,042 residents aged 16 + years). Data included measures of subjective and objective social isolation, four well-being outcomes (low life satisfaction, low feeling that life is worthwhile, low happiness and high anxiety) and participant socio-demographics, including disability status.

**Results:**

4.9% of participants were objectively socially isolated, 20.8% were subjectively socially isolated, and 7.3% were both (combined objective and subjective). Combined objective and subjective isolation related to increased likelihood of poorer outcomes in all four well-being outcomes, followed by subjective social isolation and then objective social isolation, in comparison to no social isolation. Levels of combined objective and subjective social isolation were highest in those aged 50–69, and those with a disability.

**Discussion:**

The relationship between combined objective and subjective isolation and poor well-being is stronger than for either form of social isolation alone, highlighting that individuals who experience both forms of isolation should be considered most in need of support.

## 1 Introduction

Social isolation has been highlighted as a global public health issue in need of urgent attention due to its wide-ranging effects on health and well-being and the benefits to individuals and communities afforded by stronger social networks (1). Although there are discrepancies in how social isolation and related terms are discussed and defined conceptually, social isolation is typically understood to include two related, but distinct, components: subjective social isolation and objective social isolation. Subjective social isolation refers to the perceived sense of being disconnected from others. It is often discussed in terms of loneliness – the negative emotional state that occurs when someone’s social connections are less than those they desire (1). On the other hand, objective social isolation refers to a measurable lack of connections or interactions with other people (1). This distinction is important, since it is possible to experience feelings of disconnection without being objectively socially isolated, and likewise, one could have limited social contact without necessarily feeling disconnected.

Both subjective and objective social isolation have been associated with negative physical (2, 3) and mental health outcomes (4). Evidence suggests that the relationship between subjective social isolation and poorer mental health in particular is strong; primarily with depression (5, 6) and anxiety (4, 7). Research has also reported links between subjective social isolation and broader mental health outcomes such as poorer well-being (8, 9). Well-being refers to how people feel about their day-to-day lives and functioning overall, including aspects such as life satisfaction, feeling that life is worthwhile, and positive and negative feelings such as happiness and anxiousness, respectively. Understanding these broader mental health outcomes is important, as many more individuals are affected by poor well-being than meet the criteria for clinical mental health diagnoses. Furthermore, poor well-being itself is associated with increased risk of adverse health outcomes (10).

Less is known about the relationship between objective social isolation and well-being. The literature has often focused on older adult populations (11), limiting understanding of these relationships across different life stages and highlighting an evidence gap. Moreover, studies often examine either subjective or objective social isolation, but their individual and cumulative effects have not widely been explored. Previous research has postulated that when separating the dimensions of social isolation, the subjective feelings of social isolation correspond to greatest risk of anxiety disorders (12). A study in Korea found that when people were grouped as lonely, objectively isolated, both or neither, those experiencing both loneliness and objective isolation had the highest likelihood of having a psychiatric diagnosis, followed by those who were only lonely; those who were only objectively isolated, or neither, had a much lower risk (13). These findings suggest that objective and subjective social isolation may have differing relationships with mental health (13, 14).

From a theoretical perspective, subjective and objective social isolation may have different paths to well-being. Subjective isolation (experienced as loneliness) can increase levels of stress and risk of mental health issues, both of which can lower well-being (15, 16). For example, stress brought on by the negative emotional experience of loneliness could increase overall levels of anxiety (17). Further, objective social isolation may result in poor access to practical, informational or emotional support from others, which can exacerbate existing low feelings of well-being and impede efforts to take part in activities that could improve well-being, such as engaging in paid work that would improve life satisfaction (11).

Understanding how objective and subjective isolation are related to well-being is key to designing effective interventions. Thus, this study aims to investigate how objective and subjective social isolation relate to aspects of well-being, both independently and concurrently, using a national panel survey of residents aged 16 years and over in Wales, United Kingdom (UK). With much of the current social isolation research contained to older population groups, exploration of these relationships in a population that represents a broader age range also helps address this knowledge gap.

## 2 Materials and methods

This study involved analysis of data collected through the national Time to Talk Public Health (TTPH) panel in Wales in February 2025 (18). The TTPH panel survey was established by Public Health Wales (the national public health institute in Wales) to enable regular engagement with the public to inform health practice and policy. TTPH maintains a panel of at least 2,500 residents of Wales aged 16 years and over, with a minimum target sample of 1,000 participants completing each survey. The panel is maintained and survey data collected by a market research company (MRC) procured to work with Public Health Wales.

The framework for the development, sampling and implementation of the panel is available online (18). To achieve a broadly representative survey sample, quota targets were set by NHS local health board area, age, sex, deprivation quintile (as determined by the Welsh Index of Multiple Deprivation; WIMD; (19)) and ethnicity, based on mid-2020 population estimates (20). A demographic comparison of the survey sample and Welsh population can be found in Supplementary Table S2. The initial recruitment involved a multi-step and multi-method approach, including telephone, face-to-face, social media advertising, and dissemination through networks, and panel members subsequently choose their preferred method of participation (online or telephone) for future surveys.

All panel members (*n* = 3,217) were invited to complete the February 2025 survey through their preferred method. Reminder emails and chase calls were used to fill socio-demographic gaps in the desired national population profile. In addition, social media advertising was delivered to recruit new participants to the panel and subsequently complete the survey. The advertising targeted groups typically under-represented in surveys (e.g., young people, males, ethnic minority groups). Consistent with all survey waves, to boost participation of 16–29 year olds, 100 face-to-face in-street interviews with individuals in this age group were conducted; these participants were invited to join the panel following survey completion. For the February 2025 survey, an additional 400 face-to-face in-street interviews were undertaken to address demographic gaps in the overall panel. These interviews were targeted predominantly at young males and were undertaken in more deprived areas of Wales, with sampling locations randomly selected across Health Boards proportionate to national population numbers.

This sampling approach resulted in a final survey sample of 2,137 participants: 1141 existing panel members (35% completion) and 996 new participants (507 through face-to-face interviews and 489 through social media advertising). Overall, 72% of survey participants completed the survey online, 24% through face-to-face interview and 4% by telephone.

### 2.1 Questionnaire

TTPH surveys include questions on a variety of public health topics based on organisational priorities, with questions being informed through existing validated survey questions when possible and developed in consultation with topic leads. The February 2025 survey included three questions to assess objective social isolation, one question to assess subjective social isolation, and four questions on personal well-being; all answers were self-reported. The outcome questions included in this study, their response options and data categorisation for analysis are shown in Supplementary Table S1 (21).

In summary, objective social isolation was measured using questions based on those used in similar studies using UK Biobank data (2, 3, 22) (Supplementary Table S1). Participants were asked how many people (including themselves) lived in their household; how often they visited family/friends or had family/friends visit them; and whether they engaged with any of a given list of social activities on a weekly basis (sports team or gym, pub or social club, religious group, adult education class, other). Participants were allocated one point for each of the following: living alone, visiting/having visits from friends or family less than monthly, and engaging in no weekly social activities, providing a total social isolation score out of three. Participants were categorised as objectively socially isolated if they had a total score of two or more (2, 3, 22). To measure subjective social isolation, participants were asked how connected they felt to other people in general (23). Those who responded “not at all” or “a little” were categorised as subjectively socially isolated (adapted from the measure used in The State of Social Connections report (23)).

Participants were categorised into one of four connection groups depending on their responses to the objective and subjective social isolation (SI) questions: (1) connected: not objectively or subjectively socially isolated, (2) objective SI: objectively, but not subjectively socially isolated, (3) subjective SI: subjectively, but not objectively socially isolated, (4) objective-subjective SI: both objectively and subjectively socially isolated.

Data on personal well-being was collected by asking about the following four constructs using a 10-point Likert scale (not at all to completely): life satisfaction, feeling that life is worthwhile, happiness, and anxiety. These questions were taken from the Office for National Statistics (ONS) questions on personal well-being (24) (Supplementary Table S1).

Socio-demographic data (sex, age, postcode of residence, ethnicity and disability status) for existing panel members were collected at initial panel recruitment and updated annually for accuracy. This data for new panel members was collected as a part of the survey. Sex was asked as male, female, or other, but due to low numbers the latter group was excluded from analysis (see statistical analysis section below). Age was collected using date or year of birth and was categorised into four groups (16–29, 30–49, 50–69, and 70 + years). WIMD 2019 was used by the MRC to code postcodes into deprivation quintiles (19). Ethnicity was collected using UK census categories, and due to low numbers in other than white ethnicity categories, binary codes were created (white [including white minorities], other than white). Participants were categorised as having a disability if they reported a physical or mental health condition or illness that had lasted or was expected to last 12 months or more, that reduced their ability to carry-out day-to-day activities (25). Prefer not to say was a response option for all outcome and socio-demographic questions.

### 2.2 Statistical analysis

The survey was completed by 2,137 participants. However, participants were required to have valid responses to all outcome and socio-demographic variables to be included in the analysis (i.e., all prefer not to say responses were excluded; complete case inclusion applied). This resulted in 96 participants being excluded, specifically due to responding ‘other’ or ‘prefer not to say’ to socio-demographic questions (*n* = 63), having incomplete data for objective social isolation (n = 23), subjective social isolation (*n* = 1) or well-being outcomes (*n* = 8), leaving a final sample for analysis of 2042 (Table 1).

**Table 1:** Socio-demographic breakdown of the sample and participant connection group (connected, objective SI, subjective SI, objective-subjective SI).

Socio-demographic	*n*	%	Connected	Objective SI	Subjective SI	Objective-subjective SI
All	2042		66.9%	4.9%	20.8%	7.3%
Age group (years)						
16–29	354	17.3%	79.9%	2.3%	16.7%	1.1%
30–49	724	35.5%	63.1%	4.3%	25.1%	7.5%
50–69	542	26.5%	58.7%	6.3%	22.9%	12.2%
70+	422	20.7%	73.0%	6.6%	14.2%	6.2%
*X^2^*						84.566
*p*						**<0.001**
Sex						
Male	922	45.2%	65.1%	4.7%	22.3%	7.9%
Female	1,120	54.8%	68.4%	5.2%	19.6%	6.9%
*X^2^*						3.741
*p*						0.291
Deprivation quintile						
1 - Most	297	14.5%	63.3%	5.1%	22.2%	9.4%
2	384	18.8%	64.3%	7.0%	20.6%	8.1%
3	419	20.5%	67.3%	4.8%	20.3%	7.6%
4	490	24.0%	70.6%	3.3%	18.8%	7.3%
5 - Least	452	22.1%	67.0%	5.1%	22.8%	5.1%
*X^2^*						15.589
*p*						0.211
Ethnicity						
White (including white minorities)	1958	95.9%	66.9%	4.9%	20.7%	7.5%
Other than white	84	4.1%	66.7%	6.0%	22.6%	4.8%
*X^2^*						1.114
*p*						0.774
Disability status						
With no disability	1,233	60.4%	73.8%	4.8%	18.0%	3.4%
With disability	809	39.6%	56.4%	5.2%	25.1%	13.3%
*X^2^*						99.909
*p*						**<0.001**

SI = social isolation. Bold text indicates that finding is significant, *p* < 0.05.

Chi-square tests were used to measure binary relationships between participant socio-demographics, connection groups and well-being measures. Independent relationships between outcome measures and socio-demographics were assessed using binary logistic regression (enter method). Findings from the regressions are presented as adjusted odds ratios (AORs) with 95% confidence intervals (CIs) and *p*-values. *P*-values of <0.05 were categorised as significant. Statistical analyses were conducted in SPSS v.24.

## 3 Results

Over half (54.8%) of the sample were female, 52.8% were aged 16–49 years, and 95.9% were of white ethnicity. Across deprivation quintiles, proportions ranged from 14.5% (quintile 1—most deprived) to 24.0% (quintile 4). A third (39.6%) of participants were categorised as reporting to have a disability. The socio-demographics of the overall sample are shown in Table 1.

Two thirds (66.9%) of participants were categorised as connected, 20.8% as subjective SI, 4.9% as objective SI, and 7.3% as objective-subjective SI. In chi-squared analyses, connection group was significantly related to age group and disability (Table 1). Objective-subjective SI was most common in 50–69 year olds (12.2%) and least common in 16–29 year olds (1.1%), and higher in those with a disability (13.3%) than those without a disability (3.4%).

Low life satisfaction (15.1%), low feeling that life is worthwhile (10.7%), low happiness (16.3%) and high anxiety (32.7%) were observed in the sample (Table 2). In bivariate analyses, connection group was significantly associated with all four well-being measures; those in the objective-subjective SI group consistently had the highest level of the poorest outcomes. For example, 54.7% of those in the objective-subjective SI group reported low life satisfaction, compared with just 5.6% of those in the connected group. Age group and disability were also significantly related to each well-being measure. Those aged 16–29 years reported low life satisfaction the least (9.3%), and those aged 50–69 years the most (19.2%). Further, people with disabilities reported low life satisfaction more (26.8%) than those with no disability (7.4%). Deprivation was only significantly associated with life satisfaction and feeling that life is worthwhile, while sex was only related to feelings of anxiety.

**Table 2:** Personal well-being outcomes by connection group and by participant socio-demographics.

Connection group / socio-demographics	Satisfaction				Worthwhile				Happiness				Anxiety			
	Low	Medium	High	Very high	Low	Medium	High	Very high	Low	Medium	High	Very high	High	Medium	Low	Very low
All (*n* = 2042)	15.1%	20.5%	45.7%	18.7%	10.7%	17.1%	46.1%	26.1%	16.3%	21.5%	38.9%	23.3%	32.7%	17.0%	21.6%	28.6%
Connection group																
Connected	5.6%	17.2%	53.2%	24.0%	2.8%	12.7%	51.6%	32.9%	8.3%	18.7%	43.4%	29.6%	27.5%	16.4%	24.1%	32.1%
Objective SI	10.9%	25.7%	40.6%	22.8%	5.9%	20.8%	47.5%	25.7%	13.9%	22.8%	35.6%	27.7%	33.7%	13.9%	14.9%	37.6%
Subjective SI	32.7%	29.4%	32.2%	5.6%	24.2%	27.8%	37.6%	10.4%	29.2%	29.6%	33.2%	8.0%	43.1%	19.5%	19.1%	18.4%
Objective-subjective SI	54.7%	22.0%	18.7%	4.7%	47.3%	24.7%	19.3%	8.7%	53.3%	23.3%	16.7%	6.7%	50.0%	18.0%	11.3%	20.7%
*X^2^*				486.114				513.929				356.373				83.862
*p*				**<0.001**				**<0.001**				**<0.001**				**<0.001**
Age group (years)																
16–29	9.3%	18.1%	51.7%	20.9%	5.9%	18.4%	52.0%	23.7%	13.0%	24.3%	37.6%	25.1%	28.8%	16.9%	28.5%	25.7%
30–49	17.7%	25.6%	44.1%	12.7%	12.6%	19.9%	46.1%	21.4%	19.9%	24.2%	40.5%	15.5%	40.2%	17.1%	20.6%	22.1%
50–69	19.2%	18.1%	45.9%	16.8%	13.8%	15.1%	45.0%	26.0%	18.1%	20.2%	38.6%	23.2%	30.8%	18.6%	20.3%	30.3%
70+	10.2%	17.1%	43.1%	29.6%	7.3%	14.0%	42.7%	36.0%	10.4%	16.4%	37.9%	35.3%	25.4%	14.9%	19.4%	40.3%
*X^2^*				84.384				53.278				73.909				65.449
*p*				**<0.001**				**<0.001**				**<0.001**				**<0.001**
Sex																
Male	16.2%	18.7%	46.3%	18.9%	11.3%	16.2%	46.2%	26.4%	15.9%	19.7%	38.9%	25.4%	26.7%	16.3%	24.2%	32.9%
Female	14.2%	22.2%	45.2%	18.6%	10.2%	17.9%	46.1%	25.8%	16.5%	22.9%	38.9%	21.6%	37.6%	17.7%	19.6%	25.2%
*X^2^*				4.307				1.577				5.609				34.450
*p*				0.230				0.665				0.132				**<0.001**
Deprivation quintile																
1 - Most	22.6%	20.9%	39.4%	17.2%	15.2%	19.2%	43.4%	22.2%	21.5%	19.9%	38.0%	20.5%	31.3%	19.9%	24.6%	24.2%
2	16.4%	21.6%	44.3%	17.7%	9.9%	22.9%	40.9%	26.3%	18.2%	23.2%	33.9%	24.7%	34.4%	15.4%	20.3%	29.9%
3	14.1%	18.4%	43.7%	23.9%	10.5%	13.8%	47.5%	28.2%	14.8%	20.3%	38.9%	26.0%	31.7%	16.2%	20.8%	31.3%
4	12.7%	22.7%	46.7%	18.0%	10.2%	14.5%	48.4%	26.9%	13.7%	22.2%	41.4%	22.7%	32.0%	18.6%	20.2%	29.2%
5 - Least	12.6%	19.0%	51.8%	16.6%	9.1%	16.8%	48.7%	25.4%	15.3%	21.5%	41.2%	22.1%	33.6%	15.7%	23.2%	27.4%
*X^2^*				32.897				26.597				17.106				10.299
*p*				**0.001**				**0.009**				0.146				0.590
Ethnicity																
White (including white minorities)	15.2%	20.6%	45.5%	18.8%	10.9%	17.0%	45.8%	26.4%	16.2%	21.2%	39.2%	23.3%	32.9%	17.2%	21.6%	28.3%
Other than white	13.1%	19.0%	51.2%	16.7%	6.0%	20.2%	54.8%	19.0%	17.9%	27.4%	32.1%	22.6%	27.4%	13.1%	23.8%	35.7%
*X^2^*				1.097				5.386				2.603				3.266
*p*				0.778				0.146				0.457				0.352
Disability																
With no disability	7.4%	17.3%	51.5%	23.8%	4.9%	14.2%	49.4%	31.5%	9.3%	19.6%	43.1%	27.9%	25.9%	15.8%	24.2%	34.1%
With disability	26.8%	25.5%	36.8%	10.9%	19.5%	21.6%	41.2%	17.7%	26.8%	24.4%	32.5%	16.3%	43.0%	18.9%	17.7%	20.4%
*X^2^*				205.288				157.421				139.360				88.311
*p*				**<0.001**				**<0.001**				**<0.001**				**<0.001**

SI = social isolation. Bold text indicates that finding is significant, *p* < 0.05.

Since neither connection group, nor any of the four well-being outcomes, were related to ethnicity, this variable was excluded from further analyses.

### 3.1 Multivariate relationships between connection group, socio-demographics and life satisfaction

Using binary logistical regression analysis to account for socio-demographics, compared to those in the connected group, those in the objective-subjective SI group were significantly more likely to have low life satisfaction (AOR = 14.15, *p* < 0.001), as were those in the subjective SI group (AOR 7.25; *p* < 0.001; Table 3). The objective SI group was nearly twice as likely to have low life satisfaction, but this relationship did not reach significance (AOR = 1.89, *p* = 0.069).

**Table 3:** Adjusted odds ratios (AORs) for low satisfaction, low feeling that life is worthwhile, low happiness and high anxiety by connection group and by participant socio-demographics.

Connection group / socio-demographics	Low satisfaction		Low worthwhile		Low happiness		High anxiety	
	AOR (95% CI)	*p*	AOR (95% CI)	*p*	AOR (95% CI)	*p*	AOR (95% CI)	*p*
Connection group								
Connected	*REF*	**<0.001**	*REF*	**<0.001**	*REF*	**<0.001**	*REF*	**<0.001**
Objective SI	1.89 (0.95–3.77)	0.069	2.06 (0.84–5.08)	0.115	1.72 (0.93–3.18)	0.082	1.34 (0.86–2.09)	0.198
Subjective SI	7.25 (5.26–9.97)	**<0.001**	9.64 (6.46–14.38)	**<0.001**	3.93 (2.93–5.27)	**<0.001**	1.81 (1.43–2.29)	**<0.001**
Objective-subjective SI	14.15 (9.29–21.54)	**<0.001**	21.87 (13.58–35.22)	**<0.001**	9.66 (6.48–14.39)	**<0.001**	2.20 (1.53–3.16)	**<0.001**
Age group (years)								
16–29	*REF*	0.053	*REF*	0.270	*REF*	**0.002**	*REF*	**<0.001**
30–49	1.31 (0.83–2.07)	0.242	1.27 (0.73–2.20)	0.403	1.06 (0.71–1.58)	0.768	1.22 (0.91–1.63)	0.181
50–69	1.14 (0.71–1.85)	0.584	1.10 (0.62–1.95)	0.747	0.76 (0.50–1.17)	0.214	0.82 (0.60–1.12)	0.212
70+	0.73 (0.43–1.25)	0.250	0.78 (0.41–1.48)	0.444	0.51 (0.31–0.82)	**0.006**	0.62 (0.44–0.87)	**0.005**
Sex								
Male	1.23 (0.91–1.64)	0.176	1.16 (0.83–1.63)	0.395	1.00 (0.76–1.31)	0.973	0.64 (0.52–0.78)	**<0.001**
Female	*REF*	*REF*	*REF*		*REF*		*REF*	
Deprivation quintile								
1 - Most	1.83 (1.67–2.87)	**0.009**	1.50 (0.89–2.52)	0.127	1.26 (0.83–1.91)	0.279	0.82 (0.59–1.14)	0.231
2	1.08 (0.70–1.69)	0.723	0.81 (0.48–1.36)	0.419	0.99 (0.66–1.48)	0.967	0.91 (0.67–1.23)	0.541
3	1.05 (0.67–1.63)	0.830	1.07 (0.65–1.78)	0.784	0.87 (0.58–1.31)	0.513	0.89 (0.67–1.02)	0.459
4	0.90 (0.58–1.39)	0.624	1.03 (0.63–1.69)	0.900	0.80 (0.64–1.19)	0.268	0.92 (0.69–1.22)	0.574
5 - Least	*REF*	**0.022**	*REF*	0.248	*REF*	0.283	*REF*	0.826
Disability								
With no disability	*REF*		*REF*		*REF*		*REF*	
With disability	3.66 (2.72–4.93)	**<0.001**	3.42 (2.41–4.84)	**<0.001**	2.88 (2.19–3.77)	**<0.001**	2.03 (1.66–2.49)	**<0.001**

AOR = adjusted odds ratio, REF = reference category, SI = social isolation. Bold text indicates that finding is significant, *p* < 0.05.

Participants living in areas of highest deprivation were more likely to have low life satisfaction compared to those living in the least deprived areas (AOR = 1.83, *p* = 0.009). Participants with a disability were more likely to have low life satisfaction than those with no disability (AOR = 3.66, *p* < 0.001). There were no significant relationships between life satisfaction and age group or sex.

### 3.2 Multivariate relationships between connection group, socio-demographics and feeling life is worthwhile

Compared to the connected group, likelihood of low feeling that life is worthwhile was substantially increased in the objective-subjective SI (AOR = 21.87, *p* < 0.001; Table 3) and subjective SI groups (AOR = 9.64, *p* < 0.001; Table 3). Those with disabilities were more likely to report low feeling that life is worthwhile (AOR = 3.42, *p* < 0.001). There were no significant relationships between feeling that life is worthwhile and age group, sex or deprivation.

### 3.3 Multivariate relationships between connection group, socio-demographics and happiness

Compared to the connected group, the objective-subjective SI group was considerably more likely to report low happiness (AOR = 9.66, *p* < 0.001), as was the subjective SI group (AOR = 3.93*, p* < 0.001; Table 3). People with disabilities were more likely to report low happiness than those without (AOR = 2.88, *p* < 0.001; Table 3). Compared to 16–29 year olds, those aged 70 + years were less likely to report low happiness (AOR = 0.51*, p =* 0.006). There were no significant relationships between happiness and sex or deprivation.

### 3.4 Multivariate relationships between connection group, socio-demographics and anxiety

Participants in the objective-subjective SI group (AOR = 2.20, *p* < 0.001) and the subjective SI group (AOR = 1.81*, p* < 0.001) were more likely to report high anxiety than those in the connected group (Table 3). High anxiety was more likely to be reported by participants with disabilities (v. no disabilities, AOR = 2.03*, p* < 0.001). It was less likely to be reported by males (v. females, AOR = 0.64*, p* < 0.001) and those aged 70 + years (v. 16–29 year olds, AOR = 0.62, *p =* 0.005).

## 4 Discussion

This study sought to investigate how objective and subjective social isolation relate to personal well-being, both independently and when occurring concurrently. Previous research, whilst showing a clear link between subjective forms of isolation and mental well-being (26, 27), has not expansively investigated this for objective social isolation, and little research has separated and contrasted the two constructs. Our study found that participants who were both subjectively and objectively isolated had the highest risk of all poor well-being outcomes (low life satisfaction, low feeling that life is worthwhile, low happiness, and high anxiety) compared to those experiencing neither, followed by those who were subjectively isolated only. In contrast, being objectively socially isolated (without subjective isolation) did not significantly increase the risk of any poor well-being outcome when socio-demographics were controlled for, despite reaching significance in bivariate analyses.

### 4.1 Relationship between objective and subjective social isolation and personal well-being

Our findings suggest that subjective and objective SI have a cumulative relationship with well-being that is stronger than the relationship for each individually, but also that subjective isolation has a substantially stronger relationship with well-being than objective social isolation. The differing relationship between objective and subjective social isolation and well-being reflects emerging findings in the social connection literature. As described earlier, recent research contrasted objective social isolation and subjective loneliness and found that those who experienced both were at greatest risk of psychiatric disorders, followed by those who were lonely but not objectively isolated (13). Furthermore, loneliness has been found to be more predictive of psychological outcomes (28) and anxiety levels (12) in comparison to social isolation in US samples. The present study suggests that this relationship is echoed in self-reported personal well-being, and that the greatest risk to well-being comes when an individual experiences objective and subjective isolation concurrently.

Results suggest that those who experience both subjective and objective social isolation are at significant risk of poor well-being, and that this sub-group of individuals should be considered most in need of support and intervention. A potential explanation for this finding is the impact that both subjective and objective SI have on well-being, and vice versa how poor well-being can increase isolation. For example, high anxiety may cause an individual to isolate themselves to avoid stressful situations (7), or low feeling that life is worthwhile may lead to a lack of motivation to socialise. On the other hand, feelings of loneliness can result in emotional distress, while limited social interaction can lead to limited practical support, contributing to poor well-being (26). If both objective and subjective isolation are apparent, these effects could be compounded, giving rise to a greater risk of poor well-being.

Even when social connection and all other socio-demographic variables were accounted for, disability was found to have a significant relationship with all four measures of well-being. This echoes the established finding that those with disabilities are at greater risk of poor well-being (29), an effect mediated by level of social support (30, 31), severity of disability, and employment status (32). This shows that disability in itself is a risk factor for poor well-being, which may be due to increased adversity and experience of stigma (33).

### 4.2 Prevalence of objective and subjective social isolation in the sample

Experiencing any level of disconnection was most frequently reported by those aged 50–69 years followed by those aged 30–49 years. Similarly, experiencing combined objective and subjective isolation was most frequently experienced by those aged 50–69, followed by those aged 30–49. This suggests that those in the oldest age groups were not necessarily the most socially isolated in this sample. Much of the existing research focuses on older populations, however, emerging research affirms that younger people may also be at risk of poor connections. For example, research in Wales found that social well-being was lowest in younger age groups (34). Based on our findings, future research should investigate why those in the middle age groups might experience social isolation, particularly both objective and subjective forms, as that carries greater risks of poor personal well-being.

### 4.3 Limitations

In this survey, subjective social isolation was measured using a single-item question that enquired about the level of connection people felt they had to others. It did not measure the emotional component of social isolation that could determine loneliness. Moreover, due to survey restrictions, objective social isolation was measured using a short, three question measure of isolation used in similar studies investigating the relationship between objective social isolation and health (2, 3). However, it was not a validated measure, limiting comparability of findings to those using validated measures. Neither subjective nor objective isolation measures included an explicit recall period (i.e., in the past six months), which may have introduced variation in how participants interpreted and responded to questions.

Whilst the survey sample is broadly representative, people aged 16–29, males and people living in more deprived areas were slightly under-represented (see Supplementary Table S2). The panel for TTPH is voluntary, with potential for self-selection bias, as there is no financial compensation for taking part. Participants may be particularly interested in public health topics and therefore less representative of the wider population. The survey is also cross-sectional, meaning that directions of effect or changes over time cannot be inferred from the data.

### 4.4 Future directions

The findings of this study suggest that there is a specific population, those who are both subjectively and objectively socially isolated, that are particularly at risk of poor well-being. Future research should investigate this group further by exploring why these individuals are at risk and how best they can be supported.

As this study used a measure of subjective social isolation with no emotional component, future research should incorporate a comprehensive measure of loneliness so that relationships between loneliness and well-being, and how this compares to objective isolation, can be studied in greater detail.

Furthermore, while we explored social isolation and well-being across broad age categories (16–29 years, 30–49 years, 50–69 years, 70 + years), the age profile of our sample did not enable in-depth analysis of the oldest (e.g., 85+) or youngest (e.g., 16–21) age groups. For older age groups, research suggests there are key differences in experience between those aged 70 and those of older age (35). Moreover, those of oldest age are most likely to experience disability or mobility impairments, chronic illness and poor cognitive health, including dementia, which are known to impact on an individual’s ability to connect and engage with others (36, 37). With the number of people experiencing dementia rising globally (38), understanding social isolation in this sub-group is likely to become increasingly important. Likewise, for younger age groups, those aged 16 years are likely to have different social experiences to those aged 25 years and older. As technology and social media play an increasingly central role in how young people connect and engage, further understanding of how its use relates to subjective and objective forms of isolation, both positively and negatively, would be helpful.

Finally, this study focused on the differing associations between objective and subjective isolation and personal well-being. Further research could use a similar approach to explore their associations with physical health status, as the relationship between each concept independently has been well documented but rarely compared (39, 40).

## 5 Conclusion

When analysed independently, subjective isolation has a stronger relationship with poor well-being than objective isolation. However, experiencing both subjective and objective social isolation presents a significant additional risk to personal well-being in comparison to either experience alone. This research highlights the need for increased research and intervention for this particularly at-risk group (those experiencing both objective and subjective social isolation). Furthermore, research is needed to identify interventions that can address both the objective and subjective forms of social isolation, helping to prevent the detrimental consequences of poor well-being.

## Data Availability

The raw data supporting the conclusions of this article will be made available by the authors, without undue reservation.
