# Supplementary material for: The relationship between objective and subjective social isolation and well-being: a national cross-sectional survey in Wales
Source: Front Public Health. 2026 Oct 13;14:1918382. doi: 10.3389/fpubh.2026.1918382 (PMC13710044; doi:10.3389/fpubh.2026.1918382)
Supplement: Supplementary file 1 [file Table_1.docx]

Supplementary Material

# Table S1: Variables used in the analysis

| **Outcome** | **Question** | **Response options** | **Coding** |
| --- | --- | --- | --- |
| Objective social isolation | How often do you visit friends or family or have them visit you? (Select one) | Daily  2 - 4 times a week  Weekly  Monthly  Once every 3 months  Almost never  Never | Once every 3 months = 1  Almost never = 1  Never = 1  Daily = 0  2-4 times a week = 0  Weekly = 0  Monthly = 0 |
|  | Which of the following do you engage in once a week or more often? (Select all that apply) | Sports club or gym  Pub or social club  Religious group  Adult education class  Other group activity  None of the above | None of the above = 1  Any other response = 0 |
|  | How many people (including yourself) live in your household? | 1 – 10+ | 1 (alone) = 1  2+ = 0 |
|  | Total objective social isolation score |  | Scores were summed for the above three statements.  Score of 2+ = socially isolated (1)  Score of 0 – 1 = Not socially isolated (0) |
| Subjective social disconnection | In general, how connected do you feel to people? | Not at all connected  A little connected  Fairly connected  Very connected | Not at all connected or a little connected = socially disconnected (1)  Fairly connected or very connected = not socially disconnected (0) |
| Life satisfaction | Overall, how satisfied are you with your life nowadays, where 0 is "not at all satisfied" and 10 is "completely satisfied"? | 0 (not at all satisfied) – 10 (completely satisfied) | 0 – 4 = low  5 – 6 = medium  7 – 8 = high  9 – 10 = very high |
| Life worth | Overall, to what extent do you feel that the things you do in your life are worthwhile, where 0 is "not at all worthwhile" and 10 is "completely worthwhile"? | 0 (not at all worthwhile) – 10 (completely worthwhile) | 0 – 4 = low  5 – 6 = medium  7 – 8 = high  9 – 10 = very high |
| Happiness | Overall, how happy did you feel **yesterday**, where 0 is "not at all happy" and 10 is "completely happy"? | 0 (not at all happy) – 10 (completely happy) | 0 – 4 = low  5 – 6 = medium  7 – 8 = high  9 – 10 = very high |
| Anxiety | On a scale where 0 is "not at all anxious" and 10 is "completely anxious", overall, how anxious did you feel **yesterday**? | 0 (not at all anxious) – 10 (completely anxious) | 0 – 1 = very low  2 – 3 = low  4 – 5 = medium  6 – 10 = high |

# Table S2: Demographic comparison of the survey sample and Welsh population aged 16+ years

|  | **Survey sample**  **%** | **Welsh population**  **(aged 16+ years) %** |
| --- | --- | --- |
| **Age group (years)** |  |  |
| 16-29 | 17% | 21% |
| 30-49 | 36% | 29% |
| 50-69 | 27% | 31% |
| 70+ | 21% | 19% |
| **Sex** |  |  |
| Male | 45% | 49% |
| Female | 55% | 51% |
| **Deprivation quintile** |  |  |
| 1 - Most | 15% | 19% |
| 2 | 19% | 20% |
| 3 | 21% | 21% |
| 4 | 24% | 21% |
| 5 - Least | 22% | 20% |
| **Ethnicity** |  |  |
| White | 96% | 95% |
| Other than White | 4% | 5% |

Population data retrieved from Office for National Statistics (2025): [Lower layer Super Output Area population estimates (supporting information).](https://www.ons.gov.uk/peoplepopulationandcommunity/populationandmigration/populationestimates/datasets/lowersuperoutputareamidyearpopulationestimates) Deprivation quintile data retrieved from Welsh Government (2025): [Welsh Index of Multiple Deprivation, 2025](https://www.gov.wales/welsh-index-multiple-deprivation-2025). Population estimates by ethnicity retrieved from UK Data Service (2023): [Ethnic group by age and sex at country level.](https://statistics.ukdataservice.ac.uk/ne/dataset/england-and-wales-census-2021-ethnic-group-by-age-and-sex/resource/b4490710-28b8-4e9b-bb6a-ce7a32ad1d34)
